# Foodborne Viruses and Innovative Non-Thermal Food-Processing Technologies

**DOI:** 10.3390/foods9111520

**Published:** 2020-10-23

**Authors:** Andreana Pexara, Alexander Govaris

**Affiliations:** Laboratory of Hygiene of Foods of Animal Origin, Faculty of Veterinary Science, University of Thessaly, 224 Trikalon Street, 43100 Karditsa, Greece; agovaris@vet.uth.gr

**Keywords:** foodborne viruses, high-pressure processing (HPP), cold plasma (CP), ultraviolet light (UV), irradiation, pulsed electric field (PEF)

## Abstract

In recent years, several foodborne viruses’ outbreaks have been recorded worldwide. Μost of the foodborne viruses have a low infection dose, are stable and can persist and survive in foods for a long time without loss of infectivity. The most important foodborne viruses are: human norovirus (HuNoV), human rotavirus (HRV), hepatitis A virus (HAV), hepatitis E virus (HEV), human astrovirus (HAstV), Aichi virus (AiV), sapovirus (SaV), human adenovirus (HAdV) and enterovirus (EV). In recent years, innovative non-thermal food-processing technologies including high-pressure processing (HPP), cold plasma (CP), ultraviolet light (UV), irradiation and pulsed electric field (PEF) for improving the quality and safety of foods, including foods of animal origin, have been under research. This review presents the recent data on foodborne viruses and reviews the innovative non-thermal technologies for the control of the foodborne viruses in foods.

## 1. Introduction

Among foodborne outbreaks reported worldwide, foodborne viruses’ outbreaks have increased in recent years [1]. In contrast to many microorganisms, foodborne viruses cannot grow in foods, they can survive during processing and storage of foods, and virus-contaminated foods can infect consumers.

Enteric viruses such as human noroviruses (HuNo) and hepatitis A virus (HAV) have been associated with several recorded illnesses and outbreaks, whereas a low number of human enteric viruses have caused occasional outbreaks worldwide, including human astrovirus (HAstV), human rotavirus (HRV), sapovirus (SaV), enterovirus (EV) and Aichi virus (AiV). Human enteric viruses have been reported for 13.1% and 45% of the foodborne outbreaks in the EU and the United States, respectively [2]. In addition, hepatitis E virus (HEV) is admitted as an emerging foodborne virus. In 2014, viruses were the first etiological agent (20.4%) in the foodborne outbreaks in the European Union (EU) [3]. Transmission of viruses to human is made via consumption of contaminated food, person-to-person contact or environmental reasons, e.g., water [4].

Viruses can contaminate foods at various steps of their production from pre- to post-harvest. Humans can be infected by the consumption of viral contaminated foods. However, the transmissions of viruses via contaminated foods are complicated and usually unknown [5]. A viral transmission to humans from the consumption of contaminated foods depends on various parameters such as virus stability, processing methods, the infection dose, and the susceptibility of the host [6]. Additionally, food components may protect the virus during the food processing methods and human ingestion. The dose of infection of foodborne viruses is generally low, and almost a small amount of virus can cause contamination. Foodborne viruses can survive for a long time in foods without loss of infectivity [1]. In addition, many control strategies that rely on the intrinsic and extrinsic properties of foods. e.g., pH, water activity (a_w_) for control of bacterial population in food, is ineffective against foodborne viruses [1]. Thermal processing provides an effective means for foodborne viruses inactivation, but it can change the organoleptic properties (e.g., color and texture) and reduce nutritional characteristics (e.g., protein and vitamin) of the food [7]. Nowadays, consumers’ show an increasing demand for high-quality natural food products. Since a high number of foodborne viruses’ outbreaks are related to minimally processed and ready-to-eat foods, alternative preservation methods are needed to inactivate viruses. The inactivation of viruses and the maintenance of the quality characteristics of high-risk foods are challenging for food processors [6]. Innovative, nοn-thermal food-processing technologies, including high-pressure processing (HPP), cold plasma (CP), ultraviolet light (UV), irradiation and pulsed electric field (PEF) have been examined for foodborne viruses inactivation, parallel to the maintenance of sensory and nutritional characteristic of treated food products [6,8]. It is also important to note that food processors should consider if innovative nοn-thermal food-processing technologies for inactivation of viruses can also inactivate bacterial pathogens, e.g., *Listeria monocytogenes*, that could potentially grow in the foods during cold storage.

This article presents the foodborne viruses, reviews the innovative technologies HPP, CP, UV, irradiation and PEF and focuses on their performance in inactivating viruses in foods.

## 2. Foodborne Viruses

Foodborne viruses and their characteristics are listed in Table 1. These viruses can either be RNA or DNA and single- or double-stranded. They also have different pathology.

### 2.1. Human Norovirus (HuNoV)

HuNoV, previously known as Norwalk virus, is a non-segmented positive-sense RNA, non-enveloped virus, belonging in the family Caliciviridae. Noroviruses are divided into seven genogroups (GI to GVII) with 30 genotypes found worldwide, while GI, GII and GIV are usually infecting humans. HuNoV is a human enteric pathogen and is recognized as the major etiological agent of acute gastroenteritis outbreaks worldwide; the majority of non-bacterial gastroenteritis outbreaks (90%) in the United States are associated to HuNoV [9]. In the European Union, ΗuNoV has been frequently identified in foodborne and waterborne outbreaks and has been the fourth etiological agent found in foodborne outbreaks [10].

HuNoV has a low infectious dose (<10^2^ copies/mL) and is characterized as highly contagious. Shellfish, fruits and vegetables pose a significant risk for human infection because they are consumed raw and may be contaminated with norovirus from the water environment [11].

### 2.2. Human Rotavirus (HRV)

Rotaviruses are double-stranded non-enveloped RNA viruses, belonging in the family Reoviridae [12]. They are divided into seven groups (A–G); humans are infected by groups A–C, while animals are infected by the rest of the groups. Human rotavirus (HRV) causes severe childhood gastroenteritis and diarrhea in infants and children of less than 5 years old [13]. Animal and human rotavirus discarded into sewage and the environment can contaminate surface waters, seafood, fruits and vegetables. Also, food handlers infected with rotavirus may contaminate foods. The infection dose for human is estimated at 10–100 viral particles found in food or water [9].

### 2.3. Hepatitis A Virus (HAV)

HAV is a 27 nm, non-enveloped, positive-stranded RNA virus, a member of the *Picornaviridae* family [9]. Human strains are grouped into three genotypes (I–III) and seven subgenotypes (IA–IIIB) based on their genomic characterization. The infectious dose of HAV is low, with 10–100 viral particles. During the incubation time (range 15–50 days, approximately 28 days), the virus is discarded from the body. HAV can infect humans through the fecal–oral route, direct person-to-person contact or ingestion of contaminated water or food, such as shellfish, fruits or uncooked vegetables [14].

Waterborne and foodborne HAV is estimated to infect 2–7% of the human gastroenteritis patients, and foodborne infection can often result in a larger and prolonged outbreak [9]. In the EU, HAV ranked second, after Salmonella, for the number of hospitalizations with 6.8% of all outbreak-related hospitalizations reported in 2018 [11]. It is important to know that HAV is the most heat-resistant viral pathogen [15].

### 2.4. Hepatitis E Virus (HEV)

HEV is a single-stranded, non-enveloped, icosahedral RNA virus in the family Hepeviridae, a diverse family of viruses infecting animals and human [16]. Strains of HEV belong to the Orthohepevirus genus, which is divided into four genotypes (A–D). HEV human disease is caused by genotypes A and B [17].

HEV is transmitted to humans via the fecal–oral route or the consumption of contaminated food and water. The infectious dose of HEV has not been accurately determined. HEV infection in humans can usually cause acute hepatitis. Typically, the disease is self-limiting; the mortality rates are usually high, particularly in pregnant women and in patients with preexisting liver disease, or may even evolve to chronic hepatis in immunosuppressed individuals. HEV patients may also show extra-hepatic manifestations [14].

HEV is characterized as the most common etiological agent of the acute viral hepatitis worldwide with increasing numbers of autochthonous cases observed worldwide [9]. The consumption of undercooked or raw contaminated meat from deer and boar can cause HEV in human consumers [12]. HEV is present in the meat, liver and other internal organs of infected pigs [18,19]. In addition, since large amounts of HEV are excreted in feces, animal manure used as fertilizer or runoffs can contaminate water sources with concomitant contamination of vegetables or shellfish [20].

### 2.5. Human Astrovirus (HAstV)

HAstV are small non-enveloped single-stranded positive RNA viruses belonging to the *Astroviridae* family, genus *Mamastrovirus*. Classic HAstV is grouped in 8 serotypes and is responsible for 2% to 9% of all acute nonbacterial gastroenteritis infections in the pediatric population worldwide. However, infections in immunocompromised individuals and elderly people are also reported [21]. The infection is transmitted essentially through the fecal–oral route, either directly or by ingestion of food. HAstV infection can be caused by a relatively low dose. In recent years, large foodborne HAstV outbreaks have been observed worldwide. Consumption of contaminated bivalve mollusks were usually associated in several outbreaks, due to their contamination with HAstVs in polluted water [13].

### 2.6. Aichi Virus (AiV)

AiV is a spherical (ca 30 nm in diameter) non-enveloped, single-stranded positive sense RNA genome virus classified in the genus Kobuvirus, family Picornaviridae. It was initially found in the Aichi region of Japan, in 1989, from patients suffering from gastro-enteritis infection associated with the consumption of contaminated raw oysters [22].

AiV excreted with human feces contaminates waters and it is frequently found in surface waters, in wastewater, in sewage or river water. Humans could be infected with these viruses from contaminated drinking water (after insufficient hygienic treatment) or recreational purposes contaminated water and after consumption of raw shellfish cultivated in contaminated waters [23].

### 2.7. Sapovirus (SaV)

SaV is a small (30–38 nm in diameter), positive single-stranded RNA genome virus belonging to the genus Sapovirus, and a member of the Caliciviridae family. To date, five genogroups of SaV are recognized, GI to GV. The saVsGI, GII, GIV and GV genogroups infect humans, while the GIII genogroup infects pigs [24].

Originally SaV was found to cause gastroenteritis in children, but it was also observed in gastroenteritis cases in elderly people. SaV is usually transmitted through the fecal–oral route. However, SaV can be also transmitted via contaminated drinking water and food or person-to-person contact. The infectious dose is estimated at 1015 to 2800 genomic copies [25]. SaV outbreaks have been increased recently, especially in Japan. SaV have been found in sewage (treated and untreated), river water and shellfish (oysters and clams) [24].

### 2.8. Human Adenovirus (HAdV)

HAdVs are non-enveloped, double-stranded DNA viruses that can infect humans. They belong to the genus Mastadenovirus, family Adenoviridae. HAdV causes several different clinical syndromes such as gastroenteritis, respiratory disease, hemorrhagic cystitis, hepatitis, etc. However, it is rarely associated with serious illness or death. It can affect infants and immunocompromised individuals, or patients with cardiac and pulmonary diseases [26]. HAdV is currently divided into 9 subgroups (A to I); 90 genotypes have been also recognized [27]. The serotypes causing gastroenteritis are 40–41, which belong to species F and are the most common etiological agents (5–20%) of acute gastroenteritis in young children [28].

The most common HAdV infection to humans is via the fecal–oral route. Food, particularly contaminated seafoods (shellfish), and water are also vectors of transmission and were also associated with several foodborne outbreaks [27].

### 2.9. Enterovirus (EV)

Enteroviruses (EVs) are non-enveloped, single-stranded RNA viruses, members of the Picornaviridae family. Four groups of EVs were recognized, namely, Coxsackie A, Coxsackie B, polioviruses and echoviruses, based on the clinical symptoms. Due to sequence diversity, EVs have been also classified into 15 species: rhinovirus A to C and enterovirus A to L. Three rhinovirus species (A to C) and four enterovirus species (A to D) infect millions of individuals worldwide every year [29]. They usually cause gastroenteritis infections in humans as well as meningitis and encephalitis [30].

EVs are transmitted predominantly via the fecal–oral route, but some species can be spread through respiratory secretions. The infection dose is low, 1–10 infectious viral particles. Foodborne EV outbreaks are linked to the consumption of raw shellfish, mainly oysters from a harvest area contaminated by human sewage [2].

## 3. Innovative Non-Thermal Food-Processing Technologies for the Inactivation of Foodborne Viruses

The main characteristics and the effectiveness of innovative non-thermal food-processing technologies (HPP, CP, UV, Irradiation and PEF) on the foodborne viruses’ inactivation are summarized in Table 2.

### 3.1. High-Pressure Processing (HPP)

HPP as a food preservation method can prolong the shelf life of foods by inactivating microorganisms and enzymes with minimal influence on the sensory, physical, and nutritional properties of the foods [52]. The technology applies hydrostatic pressure uniformly and instantaneously through the food product. The high pressure inactivates the microorganisms found either in the food matrix or on the surface of food [31]. Currently, the food industry in many countries has used HPP in a variety of foods products, including fresh bivalve shellfish, vegetable products, juices, beverages, jams, ready-to-eat meat products and drinkable yogurt [53]. In several countries worldwide, regulatory criteria for the safety evaluation and labeling of HPP treated foods have been set [54].

In HPP, a high pressure of ≤600 MPa at refrigerated or mild temperatures (4–45 °C) is typically applied to high moisture or liquid foods. Prior to HPP application, the foods are packaged in flexible pouches and placed in a pressure vessel filled with a liquid medium (usually water), which is used for the pressure transmission to food. The pressure is held on the product for a given exposure period that can last a few s to over 20 min, depending on the treatment method [54]. The fact that the food products are held in their final package during HPP prevents the post-pasteurization contamination. The pressure transmitting medium is also used in a next step of HPP process. As HPP is an energy-efficient process because no additional energy is needed once the desired pressure is reached, it also is considered as a relatively environmentally friendly processing technology [32,53].

HPP studies have been used for eliminating foodborne non-sporeforming bacteria, protozoa and fungi. However, other studies showed that HPP was also capable of effectively eliminating many animal and human viruses in foods [53,55]. HPP has been successfully used to inactivate viruses in high-risk foods, such as shellfish (e.g., clams and oysters) [31]. It is generally recognized that HPP denatures the capsid proteins of the virus and thus the infection virions are not able to attach and enter to the cells of the host [33]. In the case of enveloped viruses, the denature of the virion as well as the viral envelope by HPP have been also demonstrated [56].

Several studies have been highlighted on the heterogeneous nature of foodborne virus responses to HPP. Poliovirus is highly resistant to HPP, since a HPP application at 600 MPa for 1 h resulted in a 1 log virus reduction [57]. In contrast, HAV is not resistant to HPP in low pH foods, since a HPP treatment at 350 MPa for 2 min caused a 5 log virus reduction [55]. A HPP application of ≤600 MPa resulted in a high viral inactivation (≥5 log reduction) of a variety of non-enveloped viruses such as HAV, HRV, Feline calicivirus (FCV) and Murine norovirus 1 (MNV-1) used as HuNoV surrogates, and coxsackievirus A9 (CAV9), a surrogate for enteric virus [53]. Moreover, studies have revealed that the efficacy of HPP inactivation, even for the same foodborne virus, is strain-dependent [31,58,59,60].

The effectiveness of HPP for the inactivation of foodborne viruses depends on factors, such as HPP parameters (pressure, temperature, or holding time) and food characteristics (food composition, pH and water activity of foods). Chen et al. [61] examined the effect of various combinations of HPP processing parameters on FCV inactivation and found that a HPP treatment with a higher set pressure caused a higher viral inactivation compared to this of a higher increase in holding time. For instance, MNV-1 was gradually inactivated at HPP application of 350 to 450 MPa at 20 °C for 5 min, while a 450 MPa treatment resulted in a 6.9 log decrease of MNV-1 [62]. The titer of FCV was reduced by 2.8 logs after a 20 min application of 200 MPa HPP at ambient temperature, whereas the extension of the holding time to 72 min resulted in only a 0.9 log additional virus reduction [61]. HPP pressure can act either antagonistically or synergistically with temperature against viruses, while the optimal temperature for viral inactivation depends also on a specific target virus. Many viruses (e.g., HuNoV, RV, MNV, Tulane virus (TV; surrogate for HuNoV)) can easily be inactivated by HPP at refrigerated temperature (4 °C) than at room temperature [31,59,62,63]. However, certain viruses such as HAV were easily inactivated at ambient or higher temperature [62,64,65,66]. A higher inactivation of FCV has been found when pressure was applied at 4 °C in contrast to temperatures higher than 30 °C [61,62].

Among non-processing parameters, pH is a significant factor that should be taken into account when applying HPP to food products, in correlation to the virus of interest. For example, low pH significantly increased pressure inactivation of HAV in foods [65,66]. In contrast, MNV-1, FCV, TV, HuNoV and HRV were easily more inactivated by HPP in neutral than in low pH [31,55,56,63,66].

The composition of a food is important for the viral inactivation by HPP. Thus, the presence of lipids, carbohydrates, salts, proteins limits the effectiveness of HPP against viruses, and the same virus could demonstrate different pressure-resistance on different food products [33,57,65]. For example, Bovine enterovirus (BEN) and FCV, surrogates of HAV and HuNoV, respectively, were more resistant to pressure when treated in “naturally” contaminated shellfish, compared to laboratory growth media or seawater [67].

### 3.2. Cold Plasma (CP)

Cold plasma (CP) is gaining increasing scientific interest, since it is used in medicine, agriculture, environmental protection, or foods. CP is able to inactivate biological factors as viruses, bacteria, spores, yeast or fungi [68].

The term plasma is referred to designate the state of an ionized gas in chemistry and physics [69]. The CP is generated by the application of an electromagnetic or electric field to a gas. Various apparatus have been used for CP treatment of foods such as corona glow discharges, dielectric barrier discharges, radio frequencies, gliding arc discharges, atmospheric glow discharges, microwave-induced plasmas or inductively coupled plasmas [70]. The most important active species generated by plasma discharge are neutral or excited molecules and atoms, UV photons, negative and positive ions, free radicals and electrons. The presence of these active agents depends also on the plasma source, but the majority of reactive species are: vibrationally and electronically excited oxygen and nitrogen, reactive oxygen species (ROS) such as atomic oxygen O, singlet oxygen ^1^O_2_, superoxide oxygen O_2_^−^ and ozone O_3_, reactive nitrogen species (RNS) such as atomic nitrogen N, nitric oxide (^●^NO), nitrogen dioxide (^●^NO_2_) or peroxynitrite (ONOO–). Also, if humidity is high, electrically charged components such as H_2_O+, OH^•^ radical, OH– anion, or H_2_O_2_ are present. All these active compounds proved to have antimicrobial activity against various microorganisms including viruses [69].

Many research works have verified the significance of the CP in disinfection of materials in contact with food, as well as decontamination and preservation of food [68]. CP inactivation of bacteria and viruses has been applied to foods of animal origin such as meat and meat products [71] and eggshells [72]. CP can be used in foods at ambient temperature for a short treatment time, while the cost of the process is relatively low [73]. However, this method has certain disadvantages, i.e., a small working surface and poor permeability. Also, CP can result in increased lipid oxidation, loss of vitamins and organoleptic attributes, during treatment or storage [35]. The control of the quality attributes of foods and the safe application of this novel technology is essential for the commercial applications [36]. The regulatory status of CP as a food processing technology becomes increasingly important. The European Commission reported that there are no regulatory restrictions in the use of plasma as an electronic preservative method for organic foods [74].

Foodborne viruses’ inactivation by CP treatment is under intensive research. Extensive research on foodborne viruses and their surrogates have been made in aqueous and liquid media [37,75,76,77] and also on the surfaces of food [38,39,40]. The majority of the studies have been focused on the susceptibility of HuNoV [78] and its surrogates such as bacteriophage MS2 [79], FCV [80], TV and MNV [38]. Aboubakr et al. [80] reported a 6 log decrease of FCV with the application of plasma generated with 1% oxygen for 90 s, that is considerably higher as compared to other inactivation methods of FCV.

It is not clear yet how the use of CP in foods inactivates the viruses present. The studies carried out so far indicated that CP can inactivate viruses by altering their proteins, genetic components, and envelope lipids [41]. The chemical interaction of active agents, particularly reactive oxygen and nitrogen species (RONS), are crucial factors for the virucidal activity. The importance of reactive agents is dependent on various conditions such as the experimental status, the gas used for the CP application, the food matrix, the virus type and developed RONS types. These reactive species can damage the viral nucleic acid, reduce gene expression and eliminate the viral nucleic acid, or both [81]. Results from recent studies on foodborne viruses and their surrogates’ inactivation by CP revealed that the main mode of inactivation depends on virus type [42,43,74]. Thus, it is important to examine the virus components that are affected by the CP application. Additional studies are needed for the mechanisms of CP inactivation of viruses [73].

### 3.3. Ultraviolet Light (UV)

Ultraviolet light (UV) is a form of electromagnetic radiation with wavelengths from 10 nm (frequency ca 30 PHz) to 400 nm (750 THz), shorter than visible light that can damage various organisms. According to their wavelength, UV is subdivided into UV-A (315–400 nm), UV-B (280–315 nm), UV-C (200–280 nm) and vacuum UV (100–200 nm). UV-A can affect human skin color by tanning and UV-B can result in skin burning or even skin cancer. UV-C has germicidal properties and can inactivate bacteria and viruses, whereas vacuum UV (100–200 nm) is transmitted only in a vacuum and is absorbed by several substances. When UV-C photons collide with oxygen atoms, ozone is formed, which is known for its bacterial and virus inactivation activities. UV-C is rarely observed in nature due to its quick absorption [82].

UV light has been extensively studied for food safety. Compared to thermal processing, UV technology application to foods results in minimally processed products, fresh-like products with less effects on the quality characteristics of the products and microbiologically safe. UV light is also an advantageous food-processing technology, since it is a low-cost method, with no toxic or irritating by-products, and is easily applied to foods [83]. Since UV light is characterized by low penetration, the treatment was initially used in liquid foods such as milk, whey or juices, as an alternative processing method of the traditional pasteurization process. It can also be used for the disinfection of eggshells, ready-to-eat packaged meals, meat, vegetables, and food packaging surfaces [8].

The legislation of UV-treated foods for the consumers’ safety varies between countries. For example, Canada, the EU, New Zealand and Australia have almost similar approaches, and UV-treated foods are considered as novel foods. In the USA, UV light is considered by the FDA as radiation and is defined as a food additive. Since UV technology is promising in food safety, legislation should be harmonized worldwide by the globalization of UV food regulations [84].

UV radiation inactivates microorganisms by the formation of lesions and impeding DNA replication. The UV radiation can also form various DNA photoproducts such as cyclobutane pyrimidine dimers and 8-oxo-7,8-dihydro-2′-deoxyguanosine photoproducts. Thus, UV inhibits transcription and replication of nucleic acids, a situation called clonogenic death [85]. In certain cases, the metabolism can repair the DNA changes depending on the microorganism [44].

The UV efficacy depends on various factors such as UV sources, operating conditions, target microorganisms and food [44]. Different energy doses are required for causing death of various microorganisms [82]. The food composition is also crucial for the efficacy of UV light. The UV efficacy against viruses is also affected by food components such as proteins, fat, or carbohydrates [85].

Several studies have examined the resistance of various foodborne viruses to UV light. UV light predominately neutralizes the nucleic acids of the virus. However, UV applied at high doses (>1000 mW s/cm^2^) attacks the capsid proteins, resulting in the genome destabilization from the RNases present in the food [8]. The genomic RNA of enteric viruses, exposed to UV light, may also be modified. Since the repair enzymes found bacteria are not present in the viruses, the mechanism of “multiplicity of reactivation” is observed in repairing some types of affected genomes of several viruses [86]. Most of the studies on the UV dose for foodborne virus inactivation were conducted on viruses diluted in water or buffer. The UV efficiency for the inactivation of viruses depends on several factors, such as the nucleic acid of the virus strains, capsid proteins, host cell types, virus morphology and aggregation, as well as experimental conditions [8]; for example, single-stranded (ss) foodborne viruses, with different types of nucleic acid, found to be at least 10 times more susceptible to UV light than double-stranded (ds) viruses [8].

### 3.4. Irradiation

Food irradiation is a method that inactivates microorganisms in foods by using ionizing radiation from gamma rays (from the radionuclides ^60^Co or ^137^Cs), electron beams (up to 10 MeV), or X-rays (up to 5 MeV) [45]. Several international authorities reported that the irradiation technology is a safe and effective treatment for foods that can improve food safety and reduce economic losses from food spoilage [87].

However, ionizing radiation for foods applications has not been extensively used due to the reluctance of the consumers to accept irradiated food [88]. Although irradiation of food products technology is approved in approximately 60 countries, the commercial use is limited to quite few countries in Asia and North America [89]. National regulations on food irradiation vary from country to country [87]. In the USA, the FDA is authorized for permitting the use of irradiation in food [46]. Also, a regulatory framework regulates irradiated foods and food ingredients in the EU [90]. Consumers require more information about irradiated food. According to several law requirements in many countries, the use of the logo (commonly the international icon for irradiated food called the “Radura” symbol) and a statement of benefit are required in irradiated foods [91].

The irradiation is applied to foods by passing the packaged foods through a radiation chamber. Processing of the food product in the package is one of the benefits of irradiation since the possibility of post-process contamination is eliminated [45]. The irradiation of foods is advantageous since there is no loss of the quality characteristics, as indicated by several studies in many foods. However, irradiation deteriorates the sensory properties of certain foods due to the development of off-flavors and off-odors. Apart from the type of food, several factors including temperature, the package atmosphere, radiation dose, packaging material, storage time, and the food quality status before irradiation could affect the sensory alterations in irradiated foods [92].

The sources of gamma rays for foods are mainly ^60^Co, a radioactive isotope produced from ^59^Co, and ^137^Cs, a spent fuel from nuclear reactors.^60^Co is the most commonly used radionuclide in food since is characterized by a deep penetration, uniformity of dose, decay to nonradioactive nickel when spent, with a low risk to the environment, and available for use almost 95% of the emitted irradiation. However, the short half-life of 5.3 y and a slower rate of irradiation of the food compared to other irradiation sources are the main disadvantages [93].

Beta rays are a high-speed stream of electrons or positron, emitted by a radioactive atomic nucleus; due to their low energy levels, they must be accelerated to achieve the required energy for inactivating viruses in food preservation. Electron accelerators seem to present a number of advantages compared to gamma rays; the irradiation level can be changed at any time; it provides non-nuclear energy, the risk of occupational injuries is low, and can irradiate foods at a high or low dose [94].

Gamma-rays is an ionizing radiation method constituted by electronically charged atoms or molecules. The amount of gamma rays energy absorbed by the food is measured in Gray or kilo Gray (kGy). The desired dose is related to the exposure time. The quantity of energy absorbed by the food product also depends on its size, density, and thickness. The determination of the necessary doses for food processing is based on the food type and the aim of the irradiation [8]. There are specific international regulations and standards for the dosimetry used for the gamma rays irradiation of foods [95]. A 10 kGy maximum dose of gamma rays irradiation of foods was found adequate for several food types [46].

Nowadays, innovative irradiations methods such as low-energy electron beam (LEEB) and low-energy X-lay (LEEX) technologies have been developed for microbial and viral inactivation in foods [96,97]. Unlike radiation with γ-rays and X-rays, EB does not use radioisotopes, which is usually not acceptable by consumers [96].

Ionizing radiation to microorganisms can cause either direct or indirect inactivation. The most important direct effect of ionizing radiation on a microorganism is the lethal destroy to the genetic material, whether it is RNA or DNA, due to the nonspecific collision of photons of radiation with the atoms in the nucleic acids of the microorganisms. The indirect effects may be due to the free radicals formed during water radiolysis, resulting in the damage of enzymes, protein and nucleic acid. The effectiveness of irradiation is affected by radiation dose and several factors associated with the food type and composition (e.g., oxygen, water activity, and pH) and the type of microorganism [98].

Microorganisms show various resistance levels towards specific doses of irradiation [99]. Few studies have been conducted on the effects of irradiation on viruses as compared to those of bacteria. The disruption of the structure of the nucleic acid is an important factor in viral inactivation by irradiation. Due to their small size and genome, foodborne viruses are not easily inactivated by ionizing irradiation compared to fungi or bacteria [5]. Viruses survived a radiation of 12D process reduction for *Clostridium botulinum* in meat products except in previous damages by other methods [100]. The effectiveness of gamma irradiation on virus depends on many factors, including the virus size or type, food types, dose and application temperature. Early research has indicated the effect of irradiation on poliovirus in fish fillets [101], coxsackie virus in beef [98], and HRV and HAV in oysters and clams [102,103]. Sullivan et al. [102] reported that the type of food significantly affected coxsackie virus inactivation by irradiation.

### 3.5. Pulsed Electric Field (PEF)

Pulsed electric field (PEF) is a food preservation technology because it is advantageous of better retention of food quality attributes of several food products [48]. PEF-processed foods of animal origin include milk, yogurt drinks, or liquid egg products. PEF technology is advantageous due to microbiological safety and low energy requirements [49].

PEF technology provides an electrical treatment of short time (1–300 ns) with pulse electric field strength varied between 20 and 80 kV/cm to 100 to 300 V/cm [48]. PEF was not used in the food industry for several years due to inappropriate industrial equipment for food applications. To date, recent developments in pulse power generators and the improved understanding on the mechanisms involved have allowed the design of appropriate PEF equipment for commercial food applications. Thus, PEF technology equipment for the food industry has been available in the market, recently [50].

PEFs can inactivate vegetative cells of bacteria, yeast, and mold but cannot easily destroy spores. The microbial inactivation with PEF depends on several process factors (electrical field intensity, pulse wave width, temperature and time), microbiological factors (number, type and growth stage of microorganism) and food factors (acidity, antimicrobials, ions present and ionic conductivity strength). Microorganism inactivation enhances with increasing electric field intensity, process time and temperature. The temperature should be kept below 30–40 °C by providing a cooling system [104].

Studies conducted on the effect of PEF on virus inactivation in foods are rather low. It is still not known whether viruses can be inactivated in foods in contrast to laboratory media. Khadre and Yousef [49] reported that rotavirus of various concentrations was not inactivated to a PEF treatment of 20 to 29 kV/cm for 145.6 μs. This ineffectiveness of PEF against viruses in foods was attributed to the presence of the capsid proteins of the viruses as compared to the lipid membranes of bacterial cells [8].

## 4. Conclusions

Due to increased foodborne viruses’ outbreaks recorded worldwide, the development of novel processing methods for the viral inactivation in foods is important. Among several innovative non-thermal food-processing technologies for the inactivation of viruses that have been examined, HPP and CP are promising methods, while PEF or irradiation are considered less-effective methods. Further studies on the effects of innovative non-thermal food-processing technologies on viruses parallel to their effectiveness on control of other foodborne pathogens microorganisms and quality characteristics in various food are required.

## Figures and Tables

**Table 1:** Viruses transmitted via food.

Viruses	Particle/ Genome	Genus/Family	Disease	Transmission /Infection Dose	Associated Foods
Human Norovirus (HuNoV)	Non-enveloped/ssRNA	Norovirus/Caliciviridae	Acute gastroenteritis	Fecal–oral route/100 copies/mL	Shellfish oysters, fish, buffet meals, vegetables
Human Rotavirus (HRV)	Non-enveloped/segmented dsRNA	Rotavirus/Reoviridae	Viral gastroenteritis in children, adult diarrhea	Fecal–oral route, possible aerosol/10–100 infectious viral particles	Clams and oysters, fruits, vegetables
Hepatitis A (HAV)	Non-enveloped/ssRNA	Hepatovirus/Picornaviridae	Hepatitis A	Fecal–oral route/10–100 viral particles	Sandwiches, fruits, vegetables, milk, shellfish
Hepatitis E (HEV)	Non-enveloped/ssRNA	Orthohepevirus/Hepeviridae	Hepatitis E	Fecal–oral route/Unknown	Raw/undercooked boar, deer and pork meat, livers and liver sausages
Human astrovirus (HAtVs)	Non-enveloped/ssRNA	Mamastrovirus/Astroviridae	Gastroenteritis	Fecal–oral route/Unknown; relatively low	Bivalve mollusks, fruits and vegetables
Aichi virus (AiV)	Non-enveloped/ssRNA	Kobuvirus/Picornaviridae	Gastroenteritis	Fecal–oral route/Unknown	Raw shellfish
Sapovirus (SaV)	Non-enveloped/ssRNA	Sapovirus/Caliciviridae	Gastroenteritis	Fecal–oral route/Unknown; likely a low infectious dose similar to that of HuNoV	Shellfish (oysters and clams)
Human adenovirus (HAdV)	Non-enveloped/dsDNA	Mastadenovirus/Adenoviridae	Gastroenteritis, fever, respiratory disease, conjunctivitis, hemorrhagic cystitis, meningoencephalitis	Fecal–oral route; inhalation and direct contact with small droplets and contaminated surfaces/Unknown	Seafoods (shellfish)
Enterovirus (EV)	Non-enveloped/ssRNA	Enterovirus/Picornaviridae	Heart disorders hand-foot-and-mouth disease (HFMD), natal sepsis, meningitis/encephalitis	Fecal–oral predominantly Respiratory route; inhaling contaminated airborne droplets/Low; 1–10 infectious viral particles	Shellfish (mainly oysters)

ssRNA: single-stranded RNA, ssDNA: single-stranded DNA.

**Table 2:** Characteristics of food-processing technologies and effectiveness on foodborne viruses’ inactivation.

Technology	Principle	Advantages	Limitations and Drawbacks	Effectiveness on Foodborne Viruses’ Inactivation	Key Mechanism of Viruses’ Inactivation	References
High-pressure processing (HPP)	An intense pressure of ≤600 MPa at chilled or mild process temperatures (<45 °C) is held on either liquid or high-moisture-content solid foods for a given exposure period (few s to over 20 min).	Inactivation of microorganisms and enzymes. Minimal effects on nutritive and organoleptic quality. Independent of food shape or size. Uniformity of treatment throughout food. Reduced treatment times. Post-packaging treatments; prevention of the post-pasteurization contamination. Easy to use. Commercial systems available. Energy-efficient process; relatively environmentally friendly process. Positive consumer appeal. Approved by regulatory.	Foods should have >40% free water for antimicrobial effect. Efficacy depends on type of microorganism, and the food composition. Spores not inactivated. Mixed effects on enzymes. Limited packaging options. Batch processing. High cost of equipment.	Promising for viral inactivation in foods. Virus- and strain-dependent; enveloped viruses less stable than non-enveloped. Depends on processing parameters (pressure, temperature, and holding time) and non-processing parameters (food matrix, pH and water activity of foods).	Denaturation of the viral capsid proteins incapacitates the infection virions from attachment and penetration to the host cells. Enveloped viruses: distortion of the virion morphology and disruption of the viral envelope.	[8,31,32,33,34]
Cold plasma (CP)	Food are exposed to CP, which is generated by the application of an electric or electromagnetic field to a gas; various types of apparatus are used. CP consist of various active agents, radicals, reactive species, or charged particles.	High efficiency against various spoilage microorganisms and food pathogens, even sporulated. Short treatment times. No heat treatment. Relatively low cost. No shadow effects. In-package treatment.	Efficacy depends on the type of microorganism, inactivation medium, number, and physiological state of the cells. Efficacy also affected by physical and chemical properties of foods, operating gas mixture and flow. Negative effects in some of the quality attributes of the food products. Technology in an early development stage. Consideration of safe application.	Becomes a promising solution for viral inactivation in foods. Enteric viruses and their surrogates have been successfully treated in aqueous solutions, and other liquid media and also on the surfaces of food.	The main mode of inactivation depends on virus target. Chemical interaction of reactive agents, particularly ROS and RNS and charged particles. Modification and/or degradation of proteins, nucleic acids, and lipids of viral envelopes.	[31,34,35,36,37,38,39,40,41,42,43]
Ultraviolet light (UV)	An electromagnetic radiation with wavelengths (100 to 400 nm) that can induce damage in a variety of organisms. Foods are exposed to UV-C (200–280 nm): germicidal range, inactivates bacteria and viruses.	Improvement of food safety with minor effects on the nutritional and sensory properties of foods at low doses. Inactivation of bacterial spores. Equipment of moderate-to-low cost-and easy to use. Stimulates the synthesis of health-promoting compounds. Suitable for food contact surfaces.	Low degree of penetration (surface treatment). Pretreatment can be necessary. Occurrence of shadow effects. Process parameters difficult to standardize. The efficacy depends on several processed factors, target microorganisms, microbial concentration and material or food composition.	Many factors affect the efficiency, such as the type of nucleic acid of the virus, viral proteins, type of host cell, viral strain, virus aggregation, and experimental conditions. Single-stranded (ss) viruses, independent of the nucleic acid, at least 10 times more susceptible than double-stranded (ds) viruses. The food composition has great impact on efficiency.	Predominately attack of the viral nucleic acid, but at high enough doses (>1000 mW s/cm^2^) it can also affect the capsid proteins.	[8,31,34,44]
Irradiation	Packaged foods are exposed to a certain amount of ionizing radiation which mainly includes gamma rays, X-rays and electron beams.	A cold process. Highly effective. Suitable for sterilization. Insect disinfestation and parasites inactivation. Delay ripening and senescence. Excellent penetration into foods. Post-packaging treatments. Suitable for large-scale processing.	Low consumers’ preference. Expensive equipment. Possibility of affecting quality parameters. Efficacy depends on food composition and type of microorganisms. Strict safety standards.	Enteric viruses are more resistant compared to bacteria, parasites, and fungi. Many factors including the size of the virus, suspending medium/type of food product, dose and temperature affect the efficiency.	The destruction of nucleic acids. Radiolytic cleavage or crosslinking of genetic material. Formation of free radicals and other reactive species contribute to damage of nucleic acid, protein, and viral envelopes.	[8,34,45,46,47]
Pulsed electric field (PEF)	An electrical treatment of short time (from several ns to several ms) with pulse electric field strength from 100 to 300 V/cm to 20–80 kV/cm.	Minimally processing of foods; retention of sensorial, nutritional, and health-promoting attributes of some food products. Noticeably short treatment times. Improvement of energy usage economically and efficiently.	Low efficacy at destroying spores and enzymes. Other preservation techniques will be required to preserve the quality and stability of the food during distribution and storage. The industrial equipment is under development. Efficiency depends on process factors, microbial entity factors and media factors.	Doubts about the effectiveness.	Electrical breakdown of cell membranes, known as electroporation. The ineffectiveness in viruses may be explained by the presence of a protein capsid on enteric viruses compared to the lipid membranes of bacterial cells.	[8,48,49,50,51]
